# Efficacy of a hypnosis-based intervention to improve well-being during cancer: a comparison between prostate and breast cancer patients

**DOI:** 10.1186/s12885-018-4607-z

**Published:** 2018-06-22

**Authors:** C. Grégoire, H. Nicolas, I. Bragard, F. Delevallez, I. Merckaert, D. Razavi, D. Waltregny, M.-E. Faymonville, A. Vanhaudenhuyse

**Affiliations:** 1 Public Health Department and Sensation and Perception Research Group, GIGA Consciousness, University of Liège, Liège, Belgium; 2 Urology Department, CHR Citadelle, Liège, Belgium; 3 Psychology Department, University, Free University of Brussels, Brussels, Belgium; 4 Urology Department, University Hospital of Liège, University of Liège, Liège, Belgium; 5 Algology-Palliative Care Department, University Hospital of Liège, Sensation and Perception Research Group, GIGA Consciousness, University of Liège, Liège, Belgium

**Keywords:** Breast cancer, Prostate cancer, Group intervention, Hypnosis, Self-care

## Abstract

**Background:**

Prostate and breast cancer can have a lot of negative consequences such as fatigue, sleep difficulties and emotional distress, which decrease quality of life. Group interventions showed benefits to emotional distress and fatigue, but most of these studies focus on breast cancer patients. However, it is important to test if an effective intervention for breast cancer patients could also have benefits for prostate cancer patients.

**Methods:**

Our controlled study aimed to compare the efficacy of a self-hypnosis/self-care group intervention to improve emotional distress, sleep difficulties, fatigue and quality of life of breast and prostate cancer patients. 25 men with prostate cancer and 68 women with breast cancer participated and were evaluated before (T0) and after (T1) the intervention.

**Results:**

After the intervention, the breast cancer group showed positive effects for anxiety, depression, fatigue, sleep difficulties, and global health status, whereas there was no effect in the prostate cancer group. We showed that women suffered from higher difficulties prior to the intervention and that their oncological treatments were different in comparison to men.

**Conclusion:**

The differences in the efficacy of the intervention could be explained by the baseline differences. As men in our sample reported few distress, fatigue or sleep problems, it is likely that they did not improve on these dimensions.

**Trial registration:**

ClinicalTrials.gov (NCT02569294 and NCT03423927). Retrospectively registered in October 2015 and February 2018 respectively.

## Background

Prostate cancer is a major pathology in industrialized countries [1, 2] and the second leading cause of death in males [3, 4] whereas breast cancer is the most frequently diagnosed cancer and the leading cause of cancer death in females [1]. Survival rates have increased worldwide [5–7] and more and more patients are living with the consequences of cancer. These two cancers are very common, are gender specific (100% of prostate cancer patients being male, about 99% of breast cancer patients being female), and both impact the sexual organs.

Prostate cancer is frequently diagnosed in later stages because it progresses slowly, leading to delayed treatment [8]. Common treatments for prostate cancer include radical prostatectomy, radiation therapy or brachytherapy, hormonotherapy, or watchful waiting [7, 9, 10]. Treatments for breast cancer include surgery, radiation therapy, chemotherapy, and hormonotherapy [11]. These treatments can have a lot of common negative side effects on patients: pain, physical dysfunction, fatigue, sleep disturbances [12–18], as well as cognitive difficulties [19–24]. These symptoms can contribute to the development of emotional distress, mostly anxiety and depression [13, 25–29], and can postpone or impede patients’ return to work [30–32].

In addition, prostate cancer has several specific consequences, such as erectile dysfunction, loss of libido, decreased orgasmic sensation [17, 33–37], or urinary and bowel problems such as incontinence [17, 35, 38, 39]. Breast cancer also has negative impacts on women’s femininity as it alters or removes symbols of femininity such as breasts, menstruation, or fertility [40, 41]. These difficulties encountered by both prostate and breast cancer patients impact a couple’s intimacy, communication and sexuality [12, 42–46], and can persist for years after the end of treatment [47–51].

In oncology settings, several psychological interventions have been tested in order to improve some of these symptoms. Group interventions such as cognitive-behavioural therapy (CBT) and hypnosis have shown benefits for emotional distress and fatigue [52–59]. However, most of these studies focus on breast cancer patients, and prostate cancer patients are often neglected in psycho-oncological studies [7, 60]. Several systematic reviews investigated non-pharmacological interventions to improve prostate cancer patients’ well-being. In their review, Keogh et al. [61] showed that physical activity is helpful to improve general quality of life and to decrease fatigue in such patients. Other systematic reviews have also shown the benefits of physical exercise to improve quality of life [62] and fatigue [63, 64] in these patients. Larkin et al. [64] also showed the efficacy of CBT-based interventions to manage cancer-related fatigue. In their systematic review, Chambers et al. [65] showed the efficacy of CBT-based interventions to improve quality of life, psychological adjustment, and to decrease worry at a 6-month follow-up in prostate cancer patients. They also showed the positive effects of stress-management interventions on quality of life after prostate surgery. Despite these encouraging results, there is a need for more data in order to confirm the efficacy of such interventions.

Given the important negative consequences of prostate and breast cancers, it is important to design interventions to help patients to cope with the effects of treatments [34, 52, 66].

## Objectives

The aim of our longitudinal study was to test if an effective intervention for breast cancer patients could also have benefits for prostate cancer patients in terms of decreased anxiety, depression, sleep difficulties and fatigue, and increased quality of life.

## Methods

### Participants and design

#### Prostate cancer

At the end of their treatment, each eligible prostate cancer patient from two oncology services (CHU Liège and CHR Citadelle, Belgium) was directly met or contacted by phone by the experimenter to be informed of the study’s aims and design. 152 eligible patients were informed about the study, of which 101 refused to participate. Reasons for refusal were “I am not interested in the proposed intervention”, “I have no time for this”, “I can manage myself”, and “it is too far from home”. Five of the 51 remaining patients dropped out of the study because they no longer had the time or suffered from health complications, leaving a final sample of 46 participants. Of these, 25 agreed to participate in the group intervention, and were divided into 5 groups of 4 to 7 patients, whereas 21 did not agree to participate in the intervention because they were not interested or had no time for the intervention. However, they agreed to complete the questionnaires. These patients were included in the control group. Inclusion criteria were ≥ 18-years-old, ability to read, write and speak French, prostate cancer diagnosis, treatment with surgery and/or radiotherapy. Exclusion criteria were metastases or cancer recurrence at the moment of inclusion, and major cognitive or psychiatric disorder.

#### Breast cancer

Breast cancer patients (only from CHU Liège) were directly met or contacted by phone by the experimenter and asked to participate in a group intervention during or after their treatment. We used previously published data of patients included in self-hypnosis/self-care group interventions [52, 67]. In these studies, patients could choose between yoga, cognitive-behavioural therapy, or self-hypnosis/self-care groups. In this study, we focused on patients included in the self-hypnosis/self-care group. Of 426 eligible patients contacted, 114 patients were included in the study. Most common reasons for refusal were “I am not interested in the proposed intervention”, “I have no time for this”, “I can manage myself” and “it is too far from home”. Fifteen patients dropped out of the study, mostly because they no longer had the time, they did not like the intervention, or they developed health complications. Sixty-eight of the 99 remaining patients chose to participate in the hypnosis group and were divided into 13 groups of 3 to 8 participants. Twenty-four patients who did not agree to participate in any group were recruited to form the control group. Inclusion criteria were ≥ 18-years-old, ability to read, write and speak French, breast cancer diagnosis. Exclusion criteria were metastases or cancer recurrence at the moment of inclusion, benefiting from palliative care, and major cognitive or psychiatric disorder.

All participants had to complete an informed consent before starting the study.

The differences in the sample sizes are due to some recruitment difficulties encountered only for the prostate cancer patients. No a priori sample size calculation was performed before the study.

### Intervention

Self-hypnosis/self-care intervention included six 120-min sessions. For the prostate cancer patients, sessions were scheduled on a monthly basis, whereas for the breast cancer patients, they took place every 2 weeks. It means that for prostate cancer patients, the intervention lasted 6 months (1 session per month), and that for breast cancer patients it lasted 3 months (2 sessions per month). The sessions combined self-hypnosis exercises and self-care techniques and were developed by one of the authors (M-E.F) [68, 69]. This approach fosters engagement in activities, adaptation to the disease, its treatments and side effects, and well-being through discussions and tasks. Tasks are based on self-care techniques and address several topics such as adjusting self-expectation, improving self-esteem, assertiveness, finding one’s own personal needs and boundaries, etc. At the end of each session, a 15-min hypnosis exercise was conducted by the therapist and each participant received CDs with the different exercises to encourage at-home training [52, 67, 68]. This intervention aims to help patients to be an actor of their well-being, and we give them practical tasks to reactivate this active role in their improvement after cancer. During the duration of the study, each participant benefited from their usual oncological and medical care, and individual psychological care if needed. Patients from the control groups did not participate in the intervention and only benefited from usual care.

### Measures

Data were collected through questionnaires:

- *Medical and sociodemographic data* such as age, gender, language, family composition, professional occupation, personal history of cancer and treatment received were collected.
- *Hospital Anxiety and Depression Scale (HADS)* [70] measures anxiety (7 items) and depression (7 items) during the past week.
- *European Organization for Research and Treatment of Cancer - Quality of Life Core Questionnaire-30 (EORTC-QLCQ30)* [71] was developed to assess quality of life and incorporates 5 functional scales (physical, role, emotional, cognitive and social functioning) and 9 symptom-related items (fatigue, nausea and vomiting, pain, dyspnea, insomnia, appetite loss, constipation, diarrhea, and financial difficulties). A global health status can also be calculated. In this paper, only the fatigue scale and the global health status are used, as we focus on these variables.
- *Insomnia Severity Index (ISI)* [72] is a 7-item scale measuring the participant’s sleep complaints and the associated distress.

All questionnaires were administrated twice: before (T0) and after (T1) the intervention.

### Data analysis

All statistical analyses were performed using Statistica 13.3 (TIBCO Software Inc.). Baseline (T0) demographic, medical, and psychological data were compared between the treatment and control groups of each population to test initial group equivalency with MANOVA and Chi-square tests. To be considered for data analysis, patients had to complete the two assessments (T0 and T1). Group-by-time changes in depression, anxiety, global health status, fatigue and sleep difficulties were processed using multivariate analysis of variance with repeated measures (MANOVA), followed by post-hoc comparisons (Tukey’s HSD test). Effect sizes for standardised differences in means between times of evaluation were calculated using Cohen’s d, with interpretation as follows: “small” (< 0.20–0.50), “medium” (0.50–0.80), and “large” effect size (> 0.80) [73]. All tests were two-tailed and the results were considered to be significant at *p* < 0.05. Alpha was set at 0.05.

## Results

The average attendance rate was 5.3 sessions for prostate cancer patients and 5.4 for breast cancer patients. The demographic and medical data of the sample are displayed in Table 1.

**Table 1:** Demographic and medical data of the sample

	Breast cancer patients (*N* = 92)			Prostate cancer patients (*N* = 46)		
	Treatment group (*N* = 68)	Control group (*N* = 24)	*p*	Treatment group (*N* = 25)	Control group (*N* = 21)	***p***
Patient demographics						
Age (years)						
Mean (SD)	54.3 (10)	52.5 (6.8)	.535	64.11 (5.8)	65.7 (4.4)	.330
Range	29–72	39–65		47–73	58–75	
Cultural origin, *N* (%)						
Occidental Europe	66 (97.0)	0	NA^a^	25 (100)	21 (100)	NA^a^
Near and Middle East	1 (1.5)	0		0	0	
African	1 (1.5)	0		0	0	
Missing data	0	24 (100)		0	0	
Marital status, *N* (%)						
Single	5 (7.4)	1 (4.1)	.778	1 (4.0)	0	.544
Married/living with partner	52 (76.5)	18 (75)		20 (80.0)	16 (76.2)	
Divorced/separated/widowed	11 (16.2)	5 (20.8)		4 (16.0)	5 (23.8)	
Missing data	0	0		0	0	
Education level, *N* (%)						
Elementary school or less	0	3 (12.5)	**.001**	1 (4.00)	2 (9.5)	.557
Lower secondary school	8 (11.8)	7 (29.2)		1 (4.00)	4 (19.0)	
Upper secondary school	21 (30.9)	8 (33.3)		7 (28.00)	5 (23.8)	
Bachelor’s degree	6 (8.8)	4 (16.7)		3 (12.00)	1 (4.8)	
Master’s degree	29 (42.6)	2 (8.3)		12 (48.00)	8 (38.1)	
Post-graduate	4 (5.9)	0		1 (4.00)	1 (4.8)	
Missing data	0	0		0	0	
Employment status, *N* (%)						
Employed part or full time	9 (13.2)	8 (33.3)	.080	8 (32.00)	4 (19.0)	.344
Employed, taken time off	38 (55.9)	12 (50)		2 (8.00)	3 (14.3)	
Not employed	20 (29.4)	4 (16.7)		15 (60.00)	14 (66.7)	
Missing data	1 (1.5)	0		0	0	
Patient medical history						
Time since diagnosis (months)						
Mean (SD)	7.1 (5.1)	5.8 (5.0)	.368	6.16 (3.52)	6.00 (4.3)	.894
Range	1–27	0.5–19		1–15	2–22	
Cancer stage, *N* (%)						
0	1 (1.5)	3 (12.5)		0	0	
1	37 (54.4)	13 (54.2)		0	0	
2	22 (32.4)	3 (12.5)	**.022**	0	0	
3	5 (7.4)	0		0	0	
Missing data	3 (4.4)	5 (20.8)		25^b^	21^b^	
Surgery, *N* (%)						
Yes	68 (100)	23 (95.8)	NA^a^	25 (100)	21 (100)	NA^a^
No	0	0		0	0	
Missing data	0	1 (4.2)		0	0	
Chemotherapy (CT), *N* (%)						
CT completed	27 (39.7)	7 (29.2)	.727	0	0	NA^a^
During CT	20 (29.4)	8 (33.3)		0	0	
No CT	21 (30.9)	8 (33.3)		25 (100)	21 (100)	
Missing data	0	1 (4.2)		0	0	
Radiation therapy (RT), *N* (%)						
RT completed	30 (44.1)	6 (25)	.280	1 (4.0)	0	.354
During RT	6 (8.8)	2 (8.3)		0	0	
Not yet started	16 (23.5)	10 (41.7)		0	0	
No RT	16 (23.5)	5 (20.8)		24 (96.0)	21 (100)	
Missing data	0	1 (4.2)		0	0	
Hormonal therapy (HT), *N* (%)						
During HT	38 (55.9)	8 (33.3)	.209	2 (8.0)	1 (4.8)	.658
Not yet started	23 (33.8)	11 (45.8)		0	0	
No HT	7 (10.3)	4 (16.7)		23 (92.0)	20 (95.2)	
Missing data	0	1 (4.2)		0	0	

Bold values indicate significant difference (*p* < .05)

^a^NA (Not applicable) when missing data impeded the analysis, or when the two groups are exactly equivalent (*p* = 1)

^b^All prostate cancer patients were recruited after their surgery and none had metastases

### Impact of the intervention on emotional distress, sleep difficulties, fatigue and quality of life in women with breast cancer

Both the control and the treatment groups were similar at baseline, except for the stage of the disease and the education level (See Table 1). A multivariate analysis of variance of the variables with repeated measures for time of evaluation showed a significant effect of time (F(5) = 2.59; *p* = 0.031) and a significant group-by-time interaction effect (F(5) = 2.76; *p* = 0.023). Post-hoc comparisons revealed a decrease in anxiety (*p* = .000), depression (*p* = .001), fatigue (*p* = .003) and sleep difficulties (*p* = .018) and an increase in global health status (*p* = .020) among women with breast cancer who participated in the intervention (see Table 2). The analyses of the effect sizes revealed one medium effect size for the evolution of anxiety before and after the intervention, in the treatment group. All other effect sizes in this group were small.

**Table 2:** Evolution of the data after the intervention in each population

	Breast cancer group							
	Treatment group (N = 68)				Control group (N = 24)			
	T0	T1	Evolution (T0-T1)	Effect size	T0	T1	Evolution (T0-T1)	Effect size
	Mean (SD)	Mean (SD)	*p*	Cohen’s d	Mean (SD)	Mean (SD)	*p*	Cohen’s d
HADS – Anxiety	8.76 (4.14)	6.70 (3.58)	**.000**	0.66	7.17 (2.96)	7.58 (3.40)	.916	−0.13
HADS - Depression	5.02 (3.16)	3.84 (3.01)	**.001**	0.47	4.13 (3.72)	4.04 (3.00)	.999	0.04
EORTC – Global Health Status	59.19 (16.23)	65.40 (15.83)	**.020**	−0.38	56.94 (20.21)	58.33 (19.19)	.980	−0.07
EORTC – Fatigue	52.94 (26.05)	44.77 (21.72)	**.003**	0.41	51.85 (30.68)	46.30 (25.94)	.537	0.27
Insomnia Severity Index	12.65 (6.50)	10.60 (6.15)	**.018**	0.40	10.54 (6.73)	12.00 (5.54)	.618	−0.20
	Prostate cancer group							
	Treatment group (N = 25)				Control group (N = 21)			
	T0	T1	Evolution (T0-T1)	Effect size	T0	T1	Evolution (T0-T1)	Effect size
	Mean (SD)	Mean (SD)	*p*	Cohen’s d	Mean (SD)	Mean (SD)	*p*	Cohen’s d
HADS – Anxiety	6.50 (3.06)	4.88 (2.98)	.085	0.50	4.76 (3.59)	5.71 (3.81)	.545	−0.30
HADS - Depression	3.46 (2.47)	3.44 (2.89)	.992	0.01	4.19 (3.28)	5.05 (4.85)	.516	−0.29
EORTC – Global Health Status	67.67 (14.30)	69.33 (15.54)	.969	−0.15	64.29 (20.94)	65.48 (25.45)	.983	−0.07
EORTC – Fatigue	32.44 (12.39)	34.22 (16.01)	.876	−0.11	32.27 (27.87)	29.63 (29.47)	.908	0.14
Insomnia Severity Index	8.04 (5.98)	6.92 (5.87)	.704	0.23	6.95 (5.13)	5.86 (4.29)	.688	0.28

Bold values indicate significant effects

### Impact of the intervention on emotional distress, sleep difficulties, fatigue and quality of life in men with prostate cancer

Both the control and the treatment groups were similar at baseline (See Table 1). Multivariate analysis of variance of the variables with repeated measures for time of evaluation revealed no significant effect of time or group and no significant interaction effect in men with prostate cancer. Post-hoc comparisons showed no significant evolution of the data in each group after the intervention (see Table 2).

### Analysis of the baseline differences between women with breast cancer and men with prostate cancer

To understand these observed differences between men with prostate cancer and women with breast cancer, we conducted a multivariate analysis of variance on the baseline data from the two treatment groups. A significant effect of sex was shown (F(5) = 3.70; *p* = .004). Post-hoc comparisons revealed significant baseline difference between men with prostate cancer and women with breast cancer: women suffered from higher anxiety (*p* = .048), fatigue (*p* = .003) and sleep difficulties (*p* = .013) before the intervention, in comparison to men with prostate cancer. In addition, women were younger than men (*p* = .000) and the treatment they received differed. All men were off treatment when they were included in self-hypnosis/self-care group (surgery (*N* = 25), radiotherapy (*N* = 1), hormonotherapy (N = 2)), while the majority of women were still on treatment at the time of the study (chemotherapy (*N* = 20), radiation therapy (*N* = 6) or hormonal therapy (*N* = 38)). The detailed baseline comparisons of the two treatment groups are displayed in Table 3.

**Table 3:** Baseline differences between breast and prostate cancer patients (Treatment groups only)

	Breast cancer (*N* = 68)	Prostate cancer (*N* = 25)	Baseline comparison (*p*)
Patients’ demographics			
Age (years)			
Mean (SD)	54.3 (10)	64.11 (5.8)	**.000**
Range	29–72	47–73	
Cultural origin, *N* (%)			
Occidental Europe	66 (97.0)	25 (100)	.687
Near and Middle East	1 (1.5)	0	
African	1 (1.5)	0	
Missing data	0	0	
Marital status, *N* (%)			
Single	5 (7.4)	1 (4.00)	.879
Married/living with partner	52 (76.5)	18 (72.00)	
Divorced/separated/widowed	11 (16.2)	4 (8.00)	
Missing data	0	2 (8.00)	
Education level, *N* (%)			
Elementary school or less	0	1 (4.00)	.443
Lower secondary school	8 (11.76)	1 (4.00)	
Upper secondary school	21 (30.88)	7 (28.00)	
Bachelor’s degree	6 (8.82)	1 (4.00)	
Master’s degree	29 (42.65)	12 (48.00)	
Post-graduate	4 (5.88)	1 (4.00)	
Missing data	0	2 (8.00)	
Employment status, *N* (%)			
Employed part or full time	9 (13.2)	8 (32.00)	**.000**
Employed, taken time off	38 (55.9)	2 (8.00)	
Not employed	20 (29.4)	15 (60.00)	
Missing data	1 (1.5)		
Patients’ medical history			
Time since diagnosis (months)			
Mean (SD)	7.1 (5.1)	6.16 (3.52)	.381
Range	1–27	1–15	
Surgery, *N* (%)			
Yes	68 (100)	25 (100)	1.00
No	0	0	
Missing data	0	0	
Chemotherapy (CT), *N* (%)			
CT completed	27 (39.7)	0	**.000**
During CT	20 (29.4)	0	
No CT	21 (30.9)	25 (100)	
Missing data	0	0	
Radiation therapy (RT), *N* (%)			
RT completed	30 (44.1)	1 (4.00)	**.000**
During RT	6 (8.8)	0	
Not yet started	16 (23.5)	0	
No RT	16 (23.5)	24 (96.00)	
Missing data	0	0	
Hormonal therapy (HT), *N* (%)			
During HT	38 (55.9)	2 (8.00)	**.000**
Not yet started	23 (33.8)	0	
No HT	7 (10.3)	23 (92.00)	
Missing data	0	0	
Patients’ psychological state, *Mean (SD*)			
HADS – Anxiety	8.76 (4.14)	6.50 (3.06)	**.048**
HADS – Depression	5.02 (3.16)	3.46 (2.47)	.075
EORTC – Global Health Status	59.19 (16.23)	67.67 (14.30)	.066
EORTC – Fatigue	52.94 (26.05)	32.44 (12.39)	**.003**
Insomnia Severity Index	12.65 (6.50)	8.04 (5.98)	**.013**

Bold values indicate significant differences between the two groups

## Discussion

In this study, we compared the efficacy of a self-hypnosis/self-care group intervention to improve well-being between men with prostate cancer and women with breast cancer. Our results revealed an improvement in anxiety, depression, fatigue, sleep difficulties and global health status in women with breast cancer whereas no significant improvement was shown among men with prostate cancer.

As these results were unexpected, we decided to compare the two treatment groups at baseline. It appeared that the two populations differed at baseline on several variables: women experienced more anxiety, more fatigue, and more severe sleep difficulties. They were also younger than men. These baseline psychological differences could be explained by the fact that most women in our sample endured several treatments (surgery, chemotherapy, radiation therapy and/or hormonal therapy), whereas men mostly received only one surgical intervention. These multimodal treatments could negatively impact the women’s well-being, as they are known to cause a lot of negative secondary effects, as described above. These differences in emotional distress observed between men and women were also reported in previous studies on depressive patients [74], cancer patients [68, 75–77], gastroenterology patients [78, 79] and the general population [80].

These baseline differences between breast and prostate cancer patients could be a major explanation for our unexpected results observed after the self-hypnosis/self-care intervention. Indeed, as men in our sample did not suffer from high distress, fatigue, sleep problems or low quality of life at baseline, it is likely that their improvement on these variables is low and not significant. On the contrary, women showed high levels of anxiety, fatigue and sleep difficulties, and a lower global health status at baseline.

Our results can also be linked to the difference in the moment at which the intervention took place for men and women. Most men in our sample had already completed their treatment, where the majority of them only received surgery, but a lot of women were still being treated for cancer at the time of the intervention. It is possible that an intervention aimed at improving psychological well-being is more efficient if provided during treatment rather than afterwards, mostly because the treatments are generally highly distressing.

Our results could be explained by the men’s tendency to express higher a need for information than for psychological help, and to rarely use available psychological interventions [7, 81, 82]. According to our clinical practice, men with prostate cancer are generally convinced that their surgery will cure them and they discover its negative side effects after several weeks or months. A belief that participating in a psychological intervention will make them less masculine, weaker or more vulnerable is also common. These beliefs could explain the lack of interest in psychological interventions shown by other studies [7, 45, 83, 84]. Women with breast cancer, on the contrary, report higher psychological and support needs [7, 45, 81, 82, 85]. As our intervention did not focus on cancer and medical information, but proposed psychological support, sharing of experiences, and learning of self-care techniques and self-hypnosis exercises, it is possible that it did not address men’s needs but was more efficient in addressing women’s needs. In addition, several studies have highlighted the importance of proposing individualized approaches to help men at a psychological level, as some of them are reluctant to talk about their difficulties in group settings [86, 87].

Finally, our contrasting results could also be linked to the format of the intervention. Women participated in 6 sessions occurring twice a month, while men attended 6 monthly sessions. It is possible that the frequency of the sessions impacts the efficacy of the intervention. Men met less frequently and had to deal with their difficulties on their own for longer periods of time without the support of the group, which could impact the way they implemented the techniques and improved over time. However, we previously showed that monthly self-hypnosis/self-care learning sessions were efficient to improve the global quality of life in chronic pain patients [68, 69].

There are several limitations to our study. First, our sample is quite small, especially for the men with prostate cancer. In addition, no a priori sample size calculation was performed before starting the study. Future studies are needed, with exactly the same design of treatment, to allow a generalisation of our results. The difference in the number of patients included for each cancer could be explained by the results of Clover et al. [88]. They recruited 311 patients with different tumour localisations (including breast and prostate) and showed that the patients currently on treatment were more likely to ask for psychological help than patients not currently on treatment. In addition, women with cancer, especially younger ones, experienced a higher need for psychological help. It is then understandable that our sample includes a lot of younger, in treatment women. Second, women in the treatment group had more severe cancers than women in the control group. This could have impact our results. Finally, as explained above, the intervention was not provided to men and women with the same frequency, which can impact its efficacy, our results and their generalisation.

However, this is one of the first studies comparing the efficacy of a psychological intervention between men with prostate cancer and women with breast cancer, which is of great interest as prostate patients are rarely the focus of psycho-oncological studies [7, 60]. Therefore, our results highlight the importance of considering the gender of the participants before designing and providing an intervention in oncology settings. Our results also open different research perspectives. First, as already highlighted in the scientific literature [89], it seems essential to design different psychological interventions for cancer patients according to their gender. As our results suggest, an intervention efficient for breast cancer patients could not be pertinent for prostate cancer patients. Several studies suggested that interventions including some physical activity such as fitness training, or concrete stress management techniques, were more accepted by men with cancer and more efficient to improve their well-being [7, 45, 65, 90]. It seems important to assess the influence of the treatment trajectory on the efficacy of this intervention, as the type of treatment and the moment at which the patients participate in the intervention appear to impact our results. Then, future researches should also take into account the treatment journey of their participants before designing an intervention. Indeed, prostate cancer patients in the current study only had surgery, but not other therapy, such as chemotherapy, radiation therapy, and hormonal therapy. Therefore, these patients have probably less negative consequences such as fatigue, sleep difficulties and emotional distress, which may relate to the low efficacy of the intervention for these patients. Different strategies could be used to adapt this intervention to men with prostate cancer, such as the inclusion of concrete stress management techniques. It could also be useful to propose this intervention longer after men’s treatments, when they are more likely to experience persistent adverse effects of treatments. An individual psychological help could also be suggested before and after the surgery, as well as a few months later, as the group setting could not be the best option for men [86, 87], and the group intervention could be proposed to those who experience more emotional distress. Finally, a more robust design, with an a priori sample size calculation, identical intervention for both groups, and similar treatments in both group could also be used to test the effect of such an intervention on prostate and breast cancer patients.

## Conclusion

In conclusion, our study showed that the intervention combining self-care and self-hypnosis is efficient to improve emotional distress, fatigue, and sleep difficulties in women with breast cancer, but not in men with prostate cancer. These results could be explained by the baseline differences between those two populations, in terms of experienced symptoms, age, and treatments received. Furthermore, the format of the intervention is not exactly the same for the two populations. Finally, men are known to rarely use available psychological interventions, and to express a need for information rather than for psychological help. This could explain why our intervention did not improve their well-being. Further researches are needed in order to assess the efficacy of a hypnosis-based intervention on different populations in oncology settings. Our results highlighted the importance to consider treatments received and gender when designing such interventions.

## Abbreviation

CBT: cognitive-behavioral therapy
